# High glucose-induced IL-7/IL-7R upregulation of dermal fibroblasts inhibits angiogenesis in a paracrine way in delayed diabetic wound healing

**DOI:** 10.1007/s12079-023-00754-x

**Published:** 2023-05-22

**Authors:** Ruikang Gao, Peng Zhou, YiQing Li, Qin Li

**Affiliations:** Huazhong University of Science and Technology Tongji Medical College First Clinical College: Wuhan Union Hospital, Wuhan, China

**Keywords:** Diabetes, Wound healing, IL-7, IL-7R, ANGPTL4, Fibroblasts, Vascular endothelial cell, Paracrine

## Abstract

It is widely acknowledged that diabetes leads to slow wound healing and ulceration, and severe serious diabetic foot ulceration may result in amputation. In recent years, much emphasis has been placed on exploring diabetic wound healing to protect patients from adverse events. We recently found interleukin-7 (IL-7), a growth factor for B-cells and T-cells, and its receptor was significantly upregulated in high glucose-induced fibroblasts and skin of diabetic mice. Moreover, IL-7 stimulated fibroblasts secreted ANGPTL4, which inhibited angiogenesis of endothelial cells resulting in delayed wound healing. In our previous study, fibroblasts, endothelial cells and keratinocytes were exposed to normal glucose (5.5 mM) or high glucose (30 mM) medium for 24 h, and RNA sequencing showed that IL-7 and IL-7R were significantly upregulated in fibroblasts. To remove the effect of high glucose and explore the influence of IL-7, exogenous rMuIL-7 used to treat normal mice led to delayed wound healing by inhibiting angiogenesis. Vitro experiments revealed that IL-7-induced fibroblasts inhibited endothelial cell proliferation, migration and angiogenesis. Further experiments showed that fibroblast angiopoietin-like-4 (ANGPTL4) secretion exhibited the inhibitory effect which was blocked by culture with the corresponding neutralizing antibody. Overall, our study revealed signaling pathways associated with diabetic wound healing and provided the foothold for further studies on delayed wound healing in this patient population.

**Graphical abstract:**

Mechanism that high glucose activates IL-7-IL-7R-ANGPTL4 signal pathway in delayed wound healing. High glucose upregulates IL-7 and IL-7R in dermal fibroblasts. IL-7 stimulates dermal fibroblasts secreting Angptl4 which inhibits proliferation, migration and angiogenesis of endothelial cells in a paracrine way.
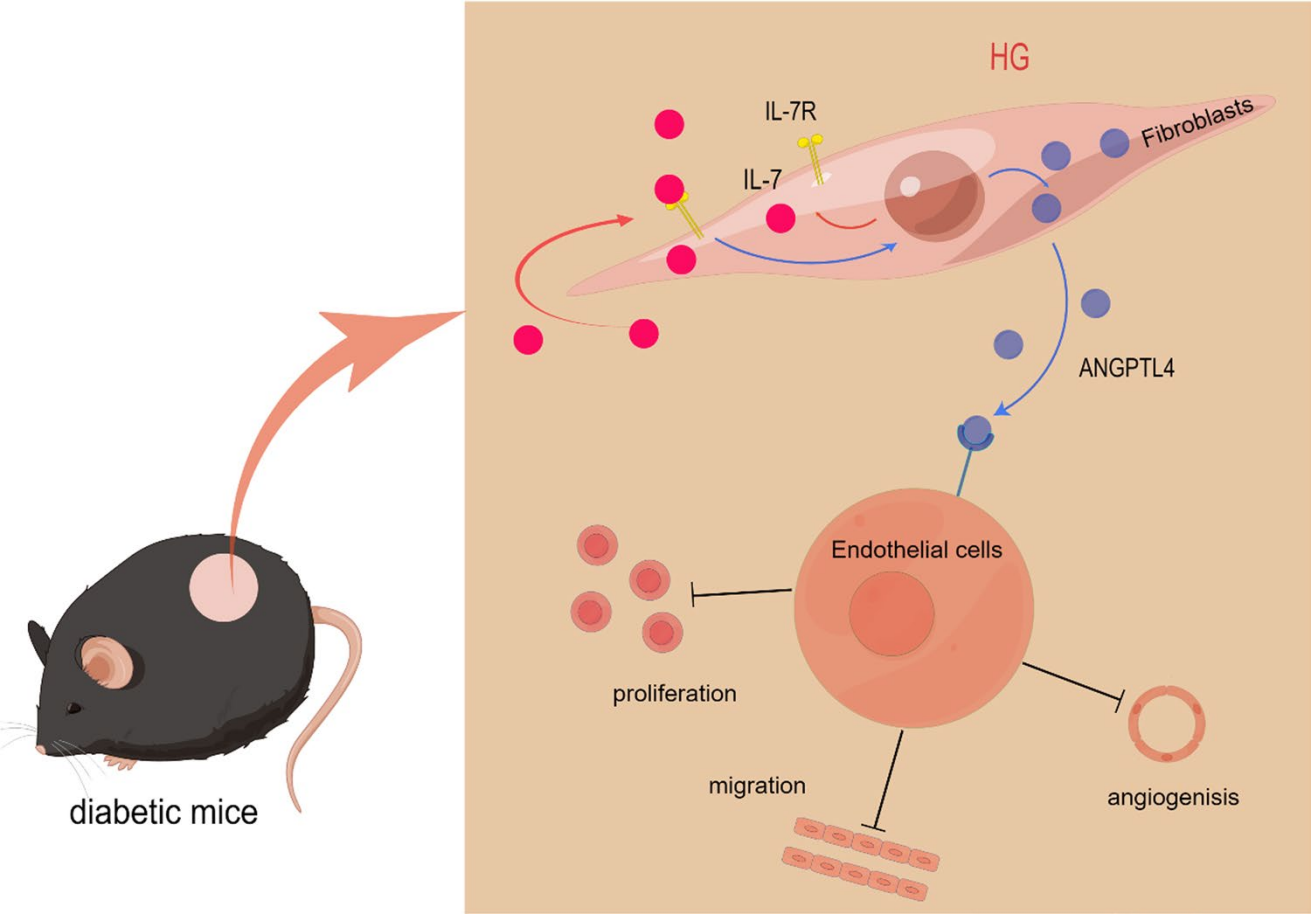

**Supplementary Information:**

The online version contains supplementary material available at 10.1007/s12079-023-00754-x.

## Introduction

Diabetes mellitus (DM) is well-established as a serious threat to global public health, causing life-threatening, disabling and costly complications regardless of the country’s socioeconomic status (Heald et al. 2020). It has been estimated that 536.6 million people have diabetes worldwide, accounting for 10.5% of 20–79-year-olds in 2021, and the prevalence is predicted to rise to 12.2% (783.2 million) in 2045. Global diabetes-related health costs are predicted to reach 966 billion USD in 2021 (Sun et al. 2021). Diabetes-related chronic wounds remain clinically challenging, emphasizing the need for further studies (Olsson et al. 2019). Wound healing is an intricate, dynamic and orderly process mediated by many cellular events, which require tight coordination to repair tissue (Rodrigues et al. 2019). It is widely thought that the wound repair process can be simplified into four main steps: hemostasis, inflammation, proliferation and dermal remodeling (Broughton et al. 2006). Little is currently known about the mechanisms underlying diabetes-related chronic wound healing, although it has been established that impaired neovascularization plays an important role.

It has been reported that vascular endothelium cells exposed to high blood glucose become dysfunctional, lose integrity and become prone to apoptosis and detachment, causing decreased vascularity and capillary density (Wilkinson and Hardman 2020; Okonkwo and DiPietro 2017). Diabetic wounds exhibit decreased vascularity and capillary density due to insufficient angiogenesis. As a result, poor angiogenesis leads to sustained tissue hypoxia and hypoperfusion, which delays wound healing (Dinh 2005).

It has been established that the human Interleukin-7 (IL-7) gene encodes a 177amino acid protein of about 18 kDa. In contrast, the murine IL-7 gene is 41 kb long and encodes for a protein of 154 amino acids protein with a molecular weight of 20 kDa (Jiang et al. 2005; McElroy et al. 2009). Current evidence suggests that Interleukin-7 is a soluble globular protein produced by the thymus, fibroblasts, stromal cells in the bone marrow and other epithelial cells, like keratinocytes and enterocytes in murine (Gray et al. 2007; Haugen et al. 2010). In humans, IL-7 has been identified in intestinal epithelial cells, keratinocytes and hepatic tissue. In addition, peripheral blood dendritic cells, follicular dendritic cells, endothelial cells, smooth muscle cells and fibroblasts can reportedly produce IL-7 (Chen et al. 2021; Lin et al. 2017). It is a hematopoietic growth factor that signals through IL-7R, a heterodimer receptor formed by IL-7Rα and a common y (Yc) chain shared with the receptors for IL-2,-4,-9,-15,-21 (Kroemer et al. 1996). An increasing body of evidence suggests that IL-7-mediated signaling initiates downstream signaling pathways through Janus kinase 1 (JAK1), JAK3, and phosphoinositide 3 kinase (PI3K), which further leads to the activation and phosphorylation of signal transducer and activator of transcription 5 (STAT5) (Chen et al. 2021; Mazzucchelli and Durum 2007).

IL-7 is well-known for its growth-promoting effects on B-cells and T-cells, and its deficiency can cause immature immune cell arrest (Oliveira et al. 2019). Overwhelming literature substantiates that both human and mouse T-cells require IL-7 for survival, proliferation, and rearrangement of some TCR genes (Carrette and Surh 2012; Kikuchi et al. 2005). In this respect, IL-7 promotes T-cell survival by upregulating the expression level of the Bcl-2 family of molecules, especially Mcl-1 and Bcl-2, which can extensively inhibit the mitochondrial apoptotic pathway (Dzhagalov et al. 2008; Jiang et al. 2004). Recently, it has been found that there is a tight correlation between IL-7 and wound healing. Annie et al. found that IL‑7 expression levels increased during chronic wound healing (Bartlett et al. 2016). Moreover, it has been reported that IL-7 enhances the differentiation of adipose-derived stem cells toward lymphatic endothelial cells through AKT signaling (Sun et al. 2019). Interestingly, IL-7 can inflame the endothelium via PI3K/AKT-dependent and independent activation of NF-kB and recruits monocytes/macrophages to the endothelium, thus playing an active role in atherogenesis (Li et al. 2012; Huang et al. 2002). However, interleukin 7 was found to upregulate vascular endothelial growth factor D (VEGF-D) in breast cancer cells and induce lymph angiogenesis in vivo (Al-Rawi et al. 2005). Besides, Osamu et al. substantiated that adding exogenous IL-7 could reduce the production of collagen I and fibronectin produced by fibroblasts. (Yamanaka et al. 2006) Taken together, these findings substantiate that IL-7 is relevant to wound healing, especially endothelium cells and fibroblasts.

Angiopoietin-like 4, ANGPLT4, is a 50 kDa secreted glycoprotein with a 15 kDa N-terminal coiled-coil domain (nANGPTL4) and a 35 kDa C-terminal fibrinogen-like domain (cANGPTL4) connected by linker region, which undergoes proteolytic processing by proprotein convertases (Aryal et al. 2019; Carbone et al. 2018). ANGPTL4 is predominantly expressed in the liver and adipose tissue and has also been detected in the vascular system and skin (Zhu et al. 2012). ANGPTL4 plays an important role in lipid metabolism by inhibiting lipoprotein lipase activity and promoting increased circulating triglyceride levels (Fernández-Hernando and Suárez 2020; Li et al. 2011). In recent years, much emphasis has been placed on the role of ANGPLT4 in angiogenesis (Dewey et al. 2016). It has been shown that tumor-derived ANGPTL4 could suppress in vitro vascular tube formation and proliferation of human umbilical vascular endothelial cells, partly due to suppression of ERK signaling (Okochi-Takada et al. 2014; Galaup et al. 2006). Another study found that C-Angptl4 could potently inhibit both bFGF- and VEGF-induced cell proliferation, migration, and tubule formation in endothelial cells and prevent neovascularization in mice by suppressing the Raf/MEK/ERK signaling cascade (Yang et al. 2008).

However, the role of IL-7 and ANGPTL4 in wound healing and their relationship remain largely unclear, warranting further research (Chong et al. 2014; Drager et al. 2013). In the present study, a significant increase in IL-7 and IL-7R was observed in high glucose-induced fibroblasts and skin of diabetic mice, suggesting activation of IL-7-IL-7R signaling. Then normal mice were treated with rMuIL-7 to explore the effect of IL-7 alone, which resulted in delayed wound healing and inhibition of angiogenesis. Human umbilical vein endothelial cells (HUVECs) were treated with rhIL-7, but the proliferation, migration and tube formation remained comparable with the control. Then rhIL-7 was used to stimulate human dermal fibroblasts (HDFs), and the supernatant was taken to culture HUVECs, resulting in HUVEC angiogenesis. Finally, we found that ANGPTL4 was the inhibitor in the supernatant and neutralizing ANGPTL4 could accelerate angiogenesis and wound healing. Our work suggests that the IL-7-IL-7R-ANGPTL4 pathway plays an important role in diabetic wound healing and has huge prospects for application as a new therapeutic target for this patient population.

## Materials and methods

### Cell and culture

Human dermal fibroblasts (HDFs) were bought from ScienCell (California, USA) and cultured in a fibroblast Medium (FM). Human umbilical vein endothelial cells (HUVECs) and human epidermal keratinocytes (HEKs) were purchased from American Type Culture Collection (ATCC) (Manassas, USA), cultured in Dulbecco’s Modified Eagle’s medium (DMEM, Gibco) containing 10% fetal bovine serum (FBS, Gibco). HDFs, HEKs and HUVECs were cultured at 37 °C with 5% CO_2_.

Normal glucose (NG) conditions consisted of DMEM containing 5.5 mM glucose, and high glucose (HG) conditions comprised of DMEM containing 30 mM glucose. The concentration of HG was achieved by adding sterile dextrose solution (4 g glucose in 20 g DEPC water, filter sterilization). D-mannitol was added in NG to elevate osmotic pressure. Different concentrations of rhIL-7 (0, 20, 40, 80 ng/mL) were added into the medium, while rMuIL-7 (100 ng/ml) was added in PBS.

### Materials and regents

Recombinant Human IL-7 (rhIL-7, Cat#: P5147-10 μg) and recombinant murine IL-7 (rMuIL-7 Cat#: P5930-10 μg) were bought from Beyotime. Mouse Angptl4 neutralizing monoclonal Antibodies were bought from Lexicon Pharmaceuticals.Human Angptl4-neutralizing monoclonal antibody was bought from Regeneron. Rabbit anti-IL-7 antibody (Cat:1650, for humans and mice) was bought from ABclonal. Rabbit anti-IL-7R antibody (Cat: 7626-1-AP, for human and mouse), rabbit anti-CD31 antibody (Cat: 28083-1-AP, for human and mouse), rabbit anti-ANGPTL4 antibody (Cat: 18374-1-AP, for human and mouse), rabbit anti-GAPDH antibody (Cat: 10494-1-AP, for human and mouse), rabbit IgG control antibody (Cat: 30000-0-AP), goat anti-rabbit IgG(H + L) HRP-conjugated Affinipure (Cat: SA00001-2), coralite488—conjugates affinipure goat anti-rabbit IgG(H + L), coralite594–conjugates affinipure goat anti-mouse IgG(H + L) were from proteintech.

### Mouse wound model and treatment

Male C57BL/6J mice (6 weeks old) were obtained from Vital River Laboratories, Beijing, and raised at the Center of Experimental Animals, Tongji Medical College, Huazhong University of Science and Technology.

Diabetic mice were established as follows: maleC57BL/6J mice (6 weeks old) were fed a high-fat diet for 4 weeks. Then the mice were fasted for 24 h and administered an intraperitoneal injection of freshly prepared streptozotocin (100 mg/kg body weight) dissolved in citric acid- sodium citrate buffer. Next, mice that exhibited polydipsia, polyuria and weight loss with blood glucose over 16 mM from the tail vein for three consecutive days indicated successful modeling (Zhang et al. 2021).

Intraperitoneal pentobarbital sodium (50 mg/kg) was used to anesthetize animals. Then the hair in the treatment region was shaved, and two 1.0 × 1.0 cm full-thickness excision skin wounds were generated on the dorsum to evaluate the effects of IL-7 or ANGPTL4 neutralizing antibody on wound healing. Digital images of the wound were captured on the day of surgery and postoperative day 1 and 3 and every other day after the third day until the wound healed. Wound areas were measured by ImageJ software (National Institutes of Health), and the wound healing rate was identified as the percentage of the initial area.

On days 7 (Fig. 2) or 11 (Fig. 8), wounded mice were euthanized, and wound tissues were paraffin-embedded, followed by staining for CD31 staining. A 15 min antigen retrieval step in citrate buffer was first conducted, and then, samples were blocked for 30 min with goat serum. Next, an anti-CD31 antibody (1:100; proteintech, Wuhan) was used to stain samples overnight at 4 °C, followed by washing with PBS and development with DAB prior to hematoxylin counterstaining.

A microscope was used to image sections, and CD31 staining was performed to assess micro-vascularization at the wound sites via quantification of the number of CD31 + cells in five random fields of view. Vessels with a 2–10 µm diameter were counted as individual vessels.

To evaluate the effects of IL-7, the normal mice received 30 µl PBS on one side and 30 µl 100 μg/ml IL-7 (dissolved in PBS) on the other side on days 0, 3, 5, 7, and 9. Moreover, the tissues were harvested on day 7 for CD31 staining.

To evaluate the effects of ANGPTL4, diabetic mice were administered IgG (0.5 mg/ml) on one side and anti-mouse ANGPTL4 (0.5 mg/ml) antibody on the other side on days 0, 3, 5, 7, 9, 11, and 13. The tissues were harvested on day 11 for CD31 staining. To establish STZ-induced diabetic models, mice were fed high-fat feed (Cat: D12108C, from New Brunswick, NJ 08,901 USA) for 4 weeks and were intraperitoneally injected with STZ (100 mg/kg) dissolved in citric acid-sodium citrate buffer once except for those in the control group. After 3 days, blood glucose levels were measured, and the mice whose blood glucose levels kept steadily over 16.7 mM for two weeks were considered diabetic (Dinh 2005) and used for the wound experiments to evaluate the effects of anti-ANGPTL4 antibody.

The Institution Animal Care and Use Committee of the Tongji Medical College, Huazhong University of Science and Technology, approved all animal studies.

### Western blotting

Cell samples or homogenized skin tissues were lysed on ice with RIPA lysis buffer according to routine procedures for protein extraction. After being centrifuged at 12,000×*g* for 10 min at 4 °C, the cleared lysate was collected, and a BCA protein assay kit was used to determine the total protein concentrations.

The collected protein was subjected to 10% or 12.5%SDS-PAGE for protein separation and transferred to PVDF membranes (Millipore, Billerica, MA, USA). The membranes were blocked in a rapid blocking solution for 20 min, followed by incubation with respective primary antibodies at 4℃ overnight. After washing with TBST (0.1%Tween-20), the membranes were incubated with the corresponding secondary antibody for 1 h at room temperature and visualized using Bio-rad Odyssey Infrared Imager.

### RNA extraction and real-time PCR

Total RNA was isolated from cells or homogenized skin tissues using TRIzol reagent following the manufacturer’s instructions. The mRNA was reverse-transcribed into cDNA using PrimeScript RT Master Mix for RT-PCR (TaKaRa). Quantitative real-time RT-PCR (qRT-PCR) was performed in triplicates on BIO-RADT100 PCR Detection System using the SYBR Green PCR Master Mix. The housekeeping gene GAPDH was used for the normalization of mRNA expression. Analysis was carried out using the 2 − ΔΔCT method. The sequences of primer pairs used are shown in Table 1.

**Table 1:** The sequences of primer pairs used in qPCR

Gene name	Sequences
Human GAPDH	Forward (5’–3’): GGAGCGAGATCCCTCCAAAAT Reverse (5’–3’): GGCTGTTGTCATACTTCTCATGG
Mouse GAPDH	Forward (5’–3’): AGGTCGGTGTGAACGGATTTG Reverse (5’–3’): TGTAGACCATGTAGTTGAGGTCA
Human IL-7	Forward (5’–3’): GGACTTCCTCCCCTGACTCT Reverse (5’–3’): TCGATGCTGACCATTAGAACACT
Mouse IL-7	Forward (5’–3’): CTGCTGCACATTTGTGGCTT Reverse (5’–3’): AAGAAGACAGGGATGCGGTG
Human IL-7RA	Forward (5’–3’): TCCAACCGGCAGCAATGTAT Reverse (5’–3’): CACACAGGCCAAGATGACCA
Mouse IL-7RA	Forward (5’–3’): AGTGGAAATGCCCAGGATGG Reverse (5’–3’): CAGTTGCTTTCACCCCTTGC
Human ANGPTL4	Forward (5’–3’): AAACCACCAGCCTCCAGAGA Reverse (5’–3’): CCTCTCCGTACCCTTCTCCA
Mouse ANGPTL4	Forward (5’–3’): CTCTTCCAAGAAGGGGAGCG Reverse (5’–3’): GAAGTCCACAGAGCCGTTCA

#### Immunofluorescent staining and immunohistochemical analysis

The mice were randomly divided into groups and euthanized on day 9 or day 13 after wounding, and harvested wound edge tissues were fixed in 4% paraformaldehyde and subsequently embedded with paraffin or flash-frozen in liquid nitrogen for protein expression analysis.

For immunofluorescent staining analysis, the skin sections were dewaxed, hydrated and antigen restored, washed with PBS and blocked in 2% BSA for 1 h. Then, the tissue was incubated with the primary antibody (Rabbit anti-IL-7 antibody 1:100, Rabbit anti-IL-7R antibody, 1:100) at 4°Covernight and then washed with 0.1% Tween-20 in TBS (TBST) three times for 5 min each followed by staining with Alexa 488 secondary antibodies (proteintech, Wuhan) for 1 h at room temperature in the dark. After washing, the slides were treated with 1 mg/mL DAPI (Beyotime) in PBS for 5 min at room temperature to visualize the nuclei. The collected images (at least three views for each sample) were then measured using ImageJ software to analyze the fluorescence intensity of IL-7 or IL-7R in different groups relative to DAPI as an internal reference.

For the immunohistochemical assay, the sections were dewaxed, hydrated and quenched the endogenous peroxidase activity with 3% H2O2 for 10 min, and then antigen was restored. After washing with PBS three times, the slices were blocked with 2% BSA for 1 h and incubated with primary CD31 antibody (1:100) overnight at 4 °C. Subsequently, after incubating with horseradish peroxidase (HRP)–conjugated secondary antibody at room temperature for 1 h, the slices were colored with DAB substrate solution. (Risau 1997) Images were captured using an Olympus FluoView FV3000 confocal microscope (Tokyo, Japan).

#### Scratch wound migration test

HUVECs were grown to confluence in a 6-well plate, and a scratch wound migration test was performed by using a 1 mL pipette tip to scratch on the surface of cells in the middle of the 6-well plate. Cells were washed with PBS, and the corresponding culture medium was added. The wound-healing process was photographed at 0, 12, 24, and 36 h after wounding. The migration rate was quantified by calculating the wound closure area versus that of the primordial wound and quantified using ImageJ software.

#### Cell counting kit 8 (CCK8)

For the CCK-8 assay, 5 × 10^3^ HUVECs were added to 96-well plates and cultured for 0, 24, 48, or 72 h. Then, 10ul/well CCK-8 reagent was added for 2 h, and absorbance measurements were taken at 450 nm.

#### Tube formation assay

HUVECs (2 × 10^4^ per well) were added to Matrigel-coated 96-well plates, and cells were assigned to groups. Cells were cultured for 6 h, then three random fields of view were imaged with an inverted microscope. The total tube length, the number of master segments and the number of meshes were quantified using ImageJ software.

#### Sandwich ELISA for detecting ANGPTL4

HDFs were cultured with rhIL-7(0 and 40 ng/ml) for 24 h, and the sample was centrifuged for 20 min at 1000 g. After adding 100 µL of each standard and sample to the appropriate wells, the plate was incubated for 2 h at 37 °C in a humid environment. After washing 4 times, 300 µL of 1 × Wash Buffer was added per well, followed by 100 µL of 1 × Detection Antibody solution and incubated for 1 h at 37 °C in a humid environment. After washing, 100 µL of 1 × HRP-conjugated antibody was added to each well. The plate was sealed with a cover and incubated for 40 min at 37 °C in a humid environment. After washing, 100 µL of TMB substrate solution was added to each well and incubated for 20 min at 37 °C in the dark. A blue color indicated a positive reaction. 100 µL of Stop Solution was added to each well in the same order as the addition of the TMB substrate leading to a color change from blue to yellow. Immediately after adding the Stop solution, the absorbance on a microplate reader was read at a wavelength of 450 nm. The average of the duplicate readings for each standard and sample was obtained and subtracted from the average zero standard absorbance (obtained from the average of the “sd0” readings). The best-fit standard curve was determined by regression analysis using a four-parameter logistic curve fit (4-PL). As an alternative, a standard curve was constructed by plotting the mean absorbance for each standard on the y-axis against the concentration on the x-axis and drawing a best-fit curve through the points on the graph.

### RNA sequencing

#### Sample preparation and total RNA extraction

RNA-Seq was performed in primary HDFs exposed to either Control or IL-7 (80 ng/ml) medium to investigate transcriptome abundance for 24 h. RNAs from cultivated HDF cells were extracted using RNAiso Plus (Cat # 9108, TaKaRa). The extracted RNA was measured and checked for concentration, purity, and integrity of RNA on a Fragment Analyzer Bioanalyzer using a standard sensitivity RNA Analysis Kit. RNA-Seq was then performed on a commercially available service (service ID # F21FTSCCKF8180_HUMacnwT, BGI, Wuhan, China).

#### Quality control and alignment of sequencing datasets

Sequencing of cDNA libraries generated over 41 to 53 million paired reads per sample. Low-quality reads of FASTQ files were filtered using SOAPnuke software v1.5.2 to obtain clean reads. After filtering, the high-quality reads were aligned against the human reference genome (GRCh38.p12) using an RNA-Seq-spliced read mapper HISAT2 (version 2.0.4). FPKM values for gene expression levels were calculated for annotated RefSeq genes using RSEM (version 1.2.8). To estimate the heterogeneity of our samples, we performed Pearson correlation coefficient analysis and PCA using R software to map clusters within the gene expression profile from all 18 samples. Normalization and differential expression analyses were estimated from count data using DESeq2. The heat map analysis was performed with the “pheatmap” R package with default parameters selecting the DEGs after FDR-corrected p values (q values ≤ 0.05) and fold changes ≥ 1.2

#### Functional enrichment analysis

Gene Ontology (GO) and Kyoto Encyclopedia of Genes and Genomes (KEGG) enrichment analysis of DEGs was performed using the Dr. TOM approach, an online database of BGI. The hypergeometric distribution was applied during GO and KEGG functional enrichment to find significantly enriched GO terms and KEGG pathways. Subsequently, the q value was obtained. We generated bubble plots to show the significantly enriched KEGG signaling pathway and GO terms. The x-axis represents the Rich Ratio (the ratio of the selected gene number annotated to a particular item to the total gene number annotated in this item), calculated using the following formula: Rich Ratio = Candidate Term Gene Num/Term Gene Num. The y-axis represents the GO term or KEGG Pathway. The GO terms and KEGG pathways with q value ≤ 0.05 were significantly enriched.

### Statistical analysis

Results were presented as means ± SEMs with at least three independent experiments calculated using GraphPad Prisms 8.0 software. The differences among treatment groups were then assessed by student’s t-test to compare the means of two groups or one-way ANOVA for more than two groups. A *P*-value < 0.05 was statistically significant.

## Results

### IL-7 and IL-7R were increased in response to the induction of diabetes

**Fig. 1:**
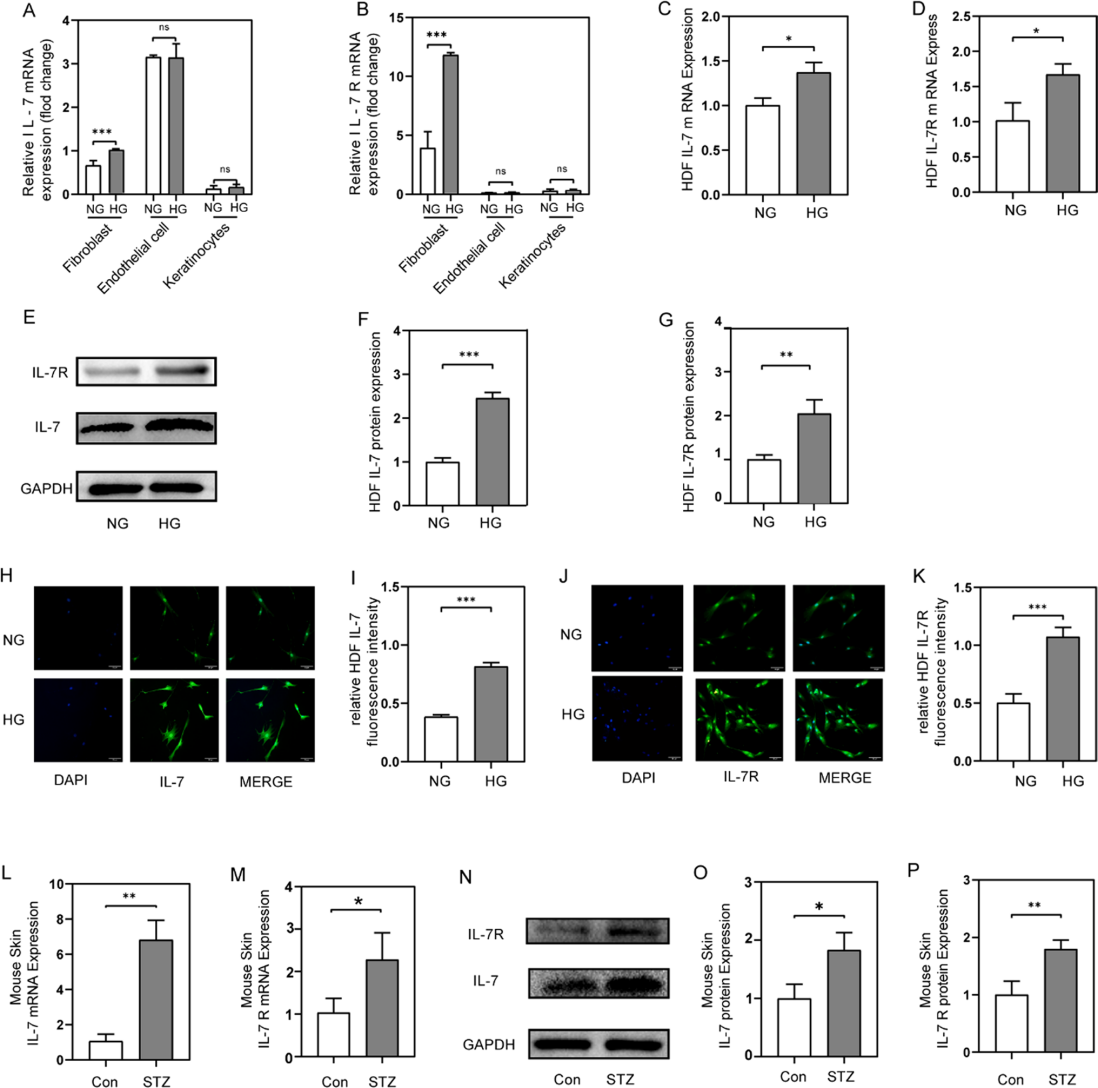
HDFs, HEKs and HUVECs were cultured in NG or HG for 24 h. Skin was acquired from normal mice and STZ induced diabetic mice which were fed for 2 weeks since blood glucose over 16 mM. **A**, **B** RNA sequencing result of IL-7 and IL-7Rin fibroblast, endothelial cells and keratinocytes in NG and HG. **C**, **D** Relative IL-7 and IL-7R mRNA expression in HDFs in NG and HG. **E**–**G** Representative western blotting image and relative quantification analysis of GAPDH, IL-7 and IL-7R in HDFs in NG and HG. **H**, **I** Representative immunofluorescent staining images and relative quantification analysis of IL-7 (green) and DAPI (blue) in HDFs in NG and HG. Scale bars, 50 mm. **J**, **K** Immunofluorescent staining images and relative quantification analysis of IL-7R (green) and DAPI (blue) in HDFs in NG and HG. Scale bars, 50 mm. **L**, **M** Relative IL-7 and IL-7R mRNA expression in skin of wild C57 and STZ induced dietetic mice. **N**–**P** Representative western blotting image and relative quantification analysis of GAPDH, IL-7 and IL-7R in skin of wild C57 and STZ induced dietetic mice. n = 3, *P < 0.05, **P < 0.01, ***P < 0.001 ns, no significance

In a previous study, we found that IL-7 and IL-7R were increased in fibroblasts, while no changes in IL-7 and IL-7R were observed in endothelial cells and keratinocytes stimulated by high glucose (30 mM) (Fig. 1A, B)(Zhang et al. 2021). Then, we examined IL-7 and IL-7R expression in HDFs treated with D-glucose. Compared with HDFs treated with mannitol, relative mRNA expression (Fig. 1C, D) and relative protein expression (Fig. 1E) of both IL-7 and IL-7R were increased. The immunofluorescent staining assay showed that IL-7 and IL-7R were expressed in the HDFs cytoplasm and, more prominently, in the nuclei. After being treated with D-glucose for 24 h, IL-7 and IL-7R were significantly upregulated in the cell cytoplasm. (Fig. 1H, J).

Streptozotocin (STZ)-induced diabetic mice were also used to investigate whether IL-7 and IL-7R participated in diabetic wound healing. We found that IL-7 and IL-7R mRNAs were significantly increased in the wound skin tissues of diabetic mice compared with non-diabetic mice (Fig. 1L, M), consistent with protein expression levels (Fig. 1N).

Therefore, we postulated that an increase of IL-7 and IL-7R may be associated with delayed healing of diabetic wounds.

### rMuIL-7 delayed normal C57BL wound healing and angiogenesis

**Fig. 2:**
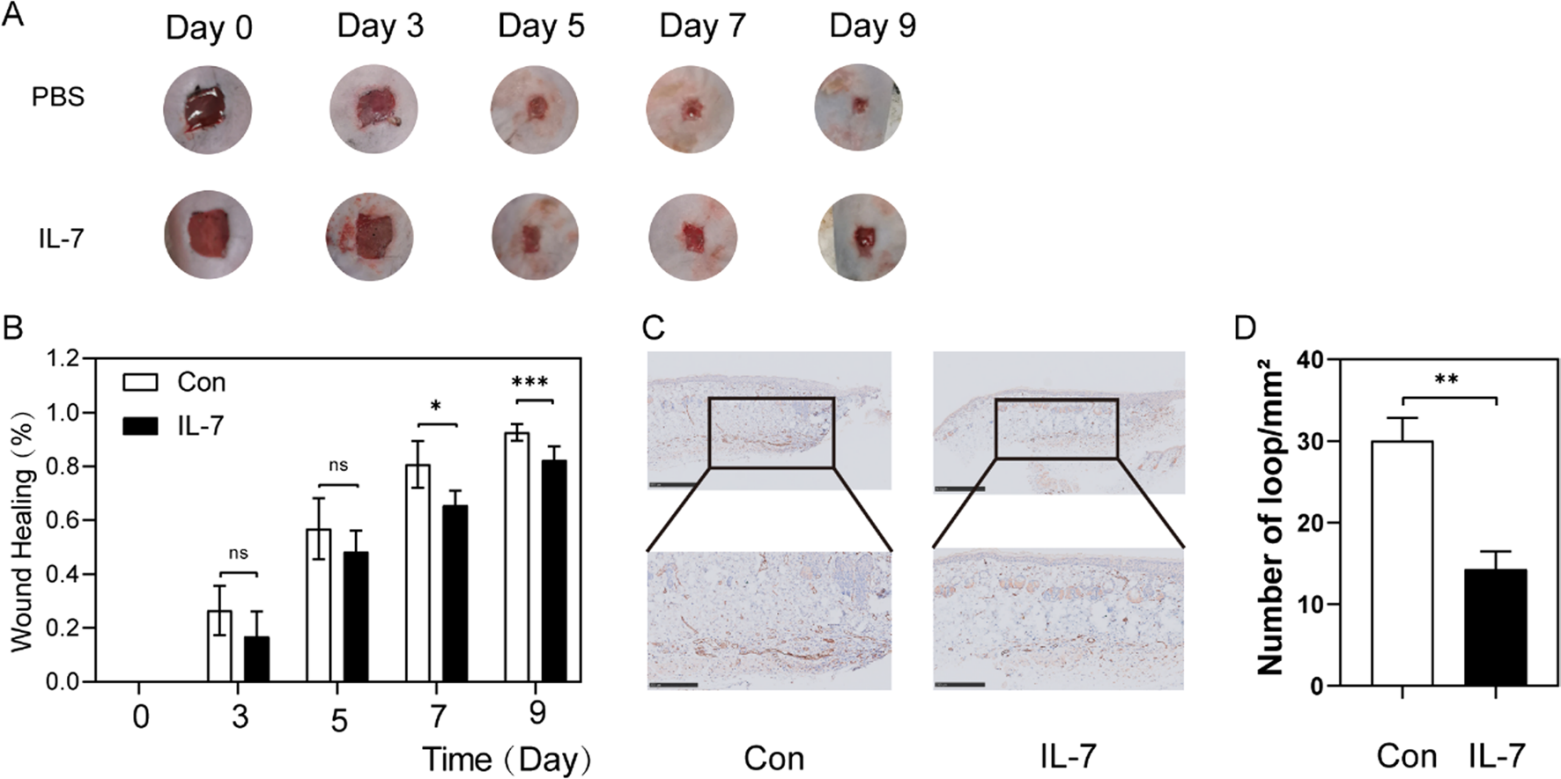
Full-thickness wounds were made on the two sides of dorsum of normal mice, and the wounds were treated locally with PBS (control side) or rMuIL-7 (IL-7 side) on days 0, 3, 5, 7 and 9 after injury. **A**, **B** Wound healing area analysis showed delayed healing in normal mice treated with rMIL-7 and the healing is shown as the percentage of the initial wound area. **A** Representative wound images at days 0, 3, 5, 7 and 9 during the healing process. Scale bars, 250 mm. **B** Quantification results of wound areas are shown (n = 3) (control side vs IL-7 side). **C** Levels of angiogenesis (vessel density) were evaluated by immunohistochemistry staining for CD31 in wound skin sections (day 7 post injury) from side of PBS, IL-7. **D** Quantification of the vessel numbers in C is presented (n = 3, three views for each sample). *P < 0.05, **P < 0.01, ***P < 0.001

It has been established that high glucose levels delay wound healing by different mechanisms (Okonkwo and DiPietro 2017). To independently explore the role of IL-7 in wound healing, we treated the wound of mice with recombinant murine Interleukin-7 (rMuIL-7, 100 ng/ml) dissolved in PBS at 100 ng/ml to preclude the influence of high glucose. Treatment with IL-7 yielded significantly delayed wound healing and closure compared with PBS (Fig. 2A, B).

Angiogenic evaluation of the wound skins from the side treated with IL-7 and PBS was carried out through immunohistochemistry staining of CD31. The results showed that the wounds treated with IL-7 exhibited poor vascularization with a nearly 53% reduction in the number of vessels in the wound bed (Fig. 2C, D), indicating impaired wound healing.

### IL-7 had no direct influence on the angiogenesis of HUVECs

Angiogenesis refers to the formation of new blood vessels based on existing blood vessels through the proliferation and migration of vascular endothelial cells based on the original capillaries (Risau 1997).

**Fig. 3:**
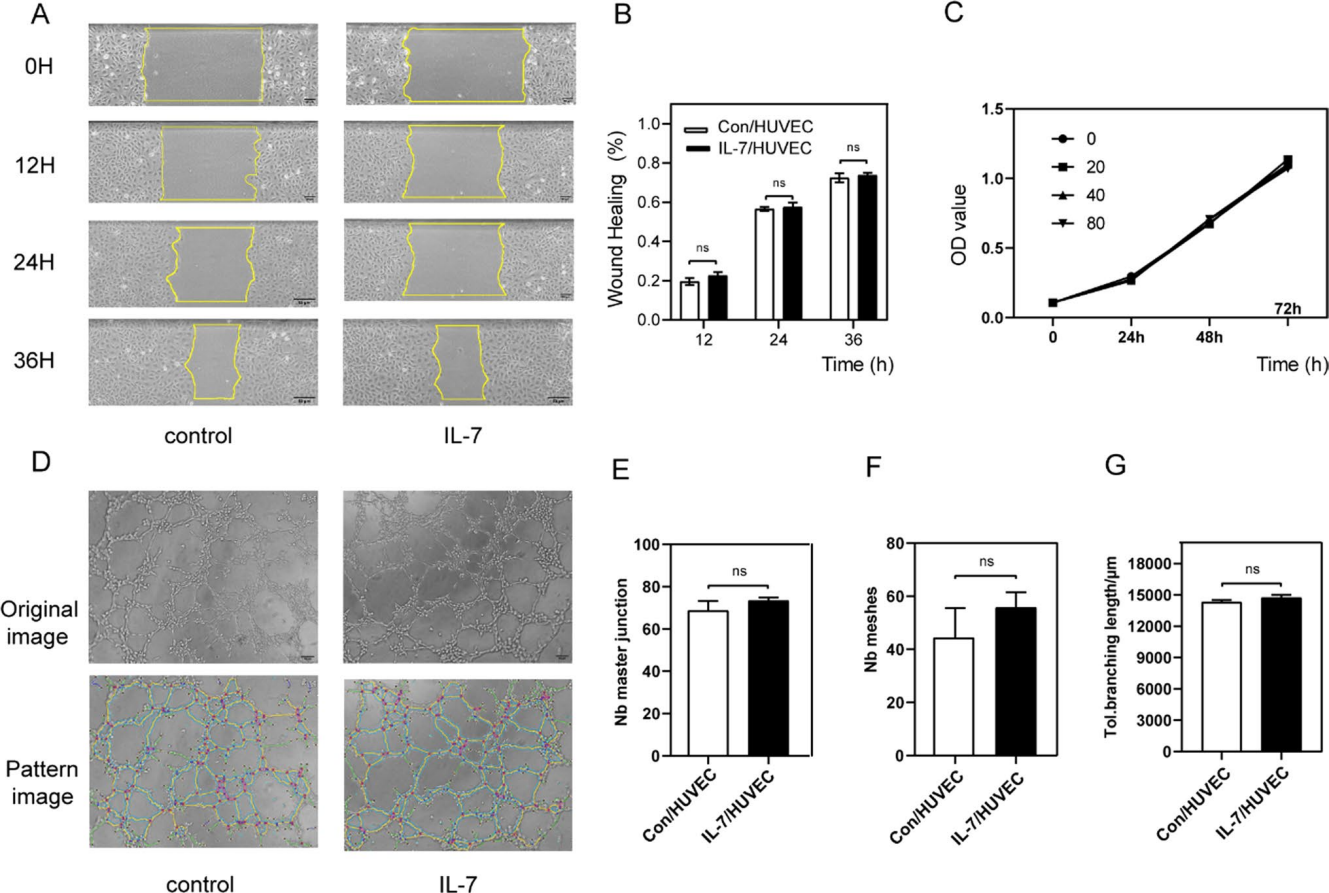
rhIL-7 directly cultured HUVECs for CCK-8 assay, scratch wound migration test and tube formation assay. **A**, **B** Scratch wound migration test image and analysis, HUVECs were treated with 0 or 40 ng/ml rhIL-7, time 0, 12, 24, 36 h Scale bars, 50 mm. **C** CCK-8 assay, HUVECs were treated with 0, 20,40,80 ng/ml rhIL-7,100ul/hole in 96-hole plate. **D**–**G** tube formation assay image and analysis; HUVECs were treated with 0 or 40 ng/ml rhIL-7; **E** number of master junction **F** number of meshes **G** total branching length,100 ul/hole in 96-hole plate, Scale bars, 50 mm. n = 3, *P < 0.05, **P < 0.01, ***P < 0.001. ns, no significance

We previously established that rMuIL-7 (100 ng/ml) could delay wound healing mediated by interfering with angiogenesis. Therefore, recombinant Human IL-7 (rhIL-7) was added to stimulated HUVECs, and the CCK-8 assay showed that rhIL-7 (0, 20, 40, 80 ng/ml) did not promote or inhibit the proliferation of HUVECs (Fig. 3C). During the scratch wound migration test, there were no differences between rhIL-7 (40 ng/ml) and control (Fig. 3A), consistent with the tube formation assay (Fig. 3D–G).

### Not IL-7 but IL-7-treated HDF secretion inhibited angiogenesis

Given that rhIL-7 had no direct influence on HUVECs, we reconsidered our RNA sequencing results. IL-7 and IL-7R was highly expressed in HDFs when treated with high glucose. We hypothesized that HDFs released IL-7, which acted on itself by combining with IL-7R. It was highly conceivable that HDF could secrete an inhibitor that acted on HUVECs, delaying wound healing and inhibiting angiogenesis.

**Fig. 4:**
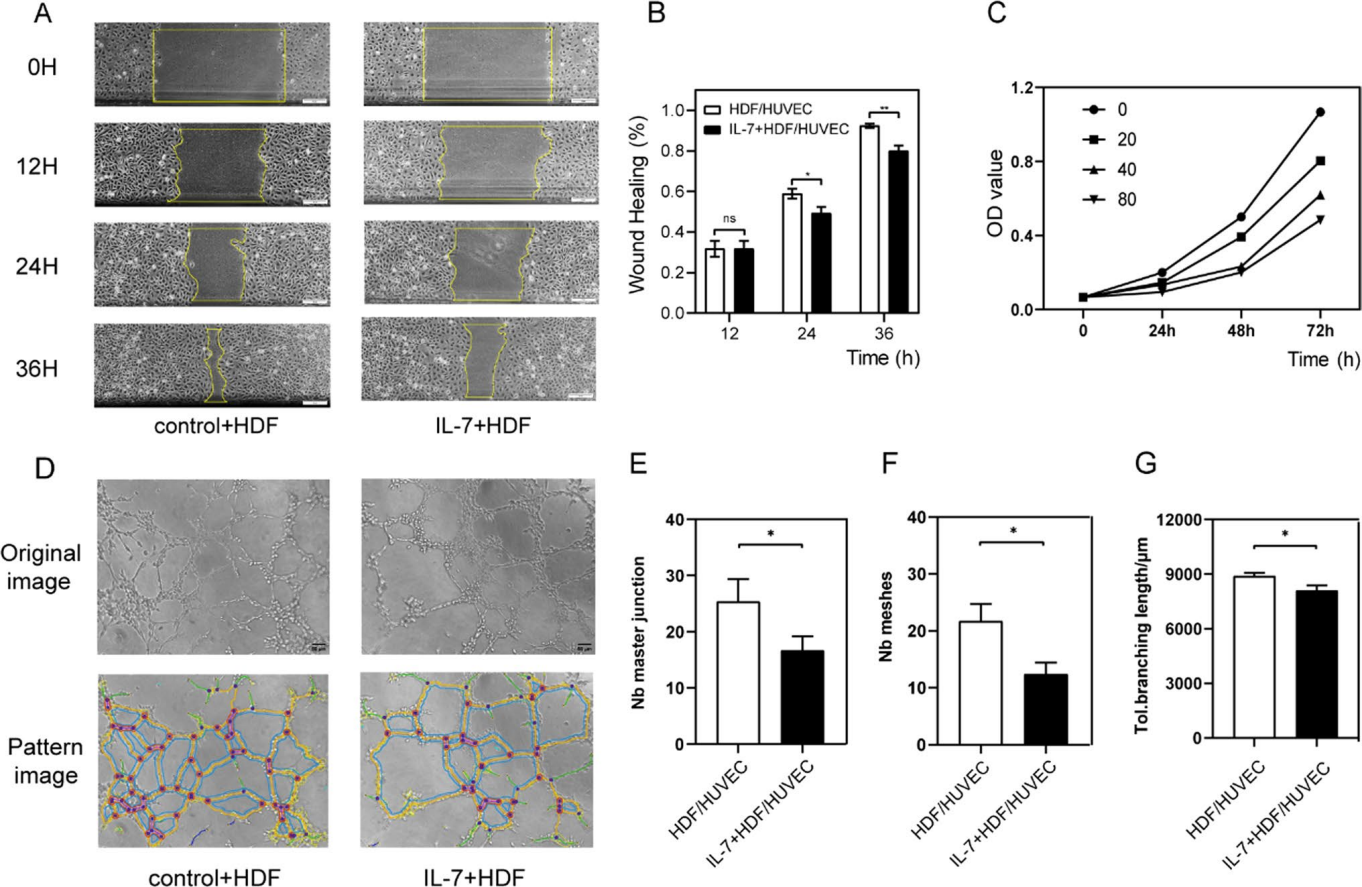
rhIL-7 stimulated HDFs for 24 h. Then supernatant was gotten to cultured HUVECs for CCK-8 assay, scratch wound migration test and tube formation assay. **A**, **B** Scratch wound migration test image and analysis, HUVECs were treated by the supernatant that cultured HDFs with 0 or 40 ng/ml rhIL-7, time 0, 12, 24, 36 h Scale bars, 50 mm. **C** CCK-8 assay, HUVECs were treated by the supernatant that cultured HDFs with 0, 20, 40, 80 ng/ml rhIL-7, tested in day 0, 1, 2, 3. **D**–**G** Tube formation assay image and analysis, HUVECs were treated by the supernatant that cultured HDFs with 0 or 40 ng/ml rhIL-7; **D** number of master junction **E** number of meshes, **F** total branching length; Scale bars, 50 mm. n = 3, *P < 0.05, **P < 0.01, ***P < 0.001

Accordingly, we used rhIL-7 to culture HDFs, and the supernatant was collected to stimulate HUVECs. As shown in Fig. 4C, the proliferation of HUVEC was gradually inhibited with increasing concentrations of rhIL-7 (0, 20, 40, 80 ng/ml). Moreover, the migration of HUVECs was restrained when the supernatant was used to stimulate HUVECs, cultured HDFs with 40 ng/ml IL-7 (Fig. 4A, B). Furthermore, the secretion also decreased the total tube length, the number of master segments and the number of meshes (Fig. 4D–G). Thus, IL-7-treated HDFs secretions damaged the function of endothelial cells in vitro.

### RNA sequencing showed gene and signal pathway changes induced by IL-7 in HDFs

**Fig. 5:**
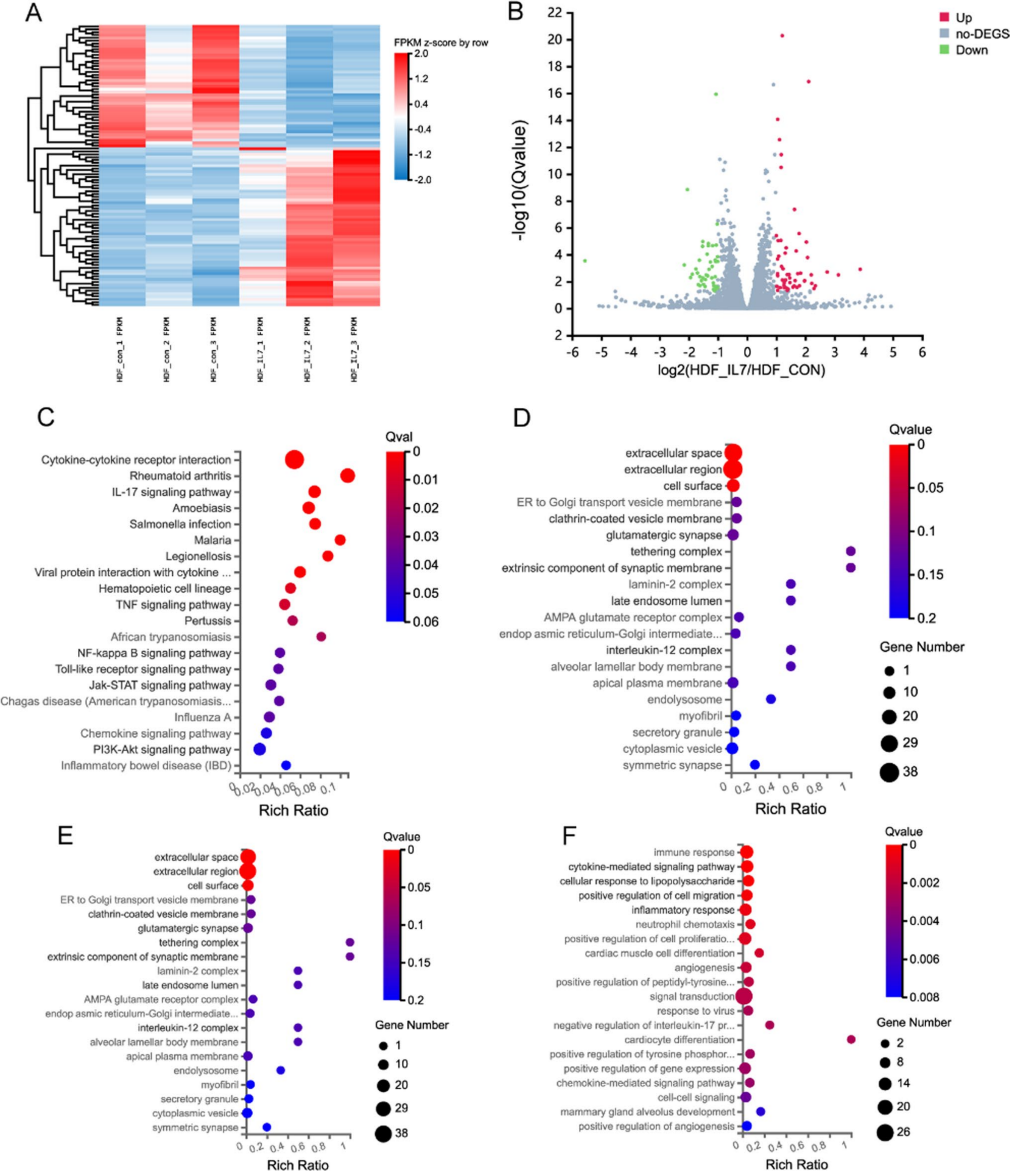
Bioinformatics analysis of DEGs in HDFs following 40 ng/ml rhIL-7 stimulation. **A** Heat map of 99 genes that were differentially expressed between HUVEC_CON (n = 3) and HUVEC_IL-7 (n = 3). Red and blue represent high and low expression, respectively. **B** Volcano plots representing up- and downregulated DEGs of HDFs in response to stimulation conditions. The size of each data point has been plotted against the negative log10 of the q value × log^2^ of the fold change, q value ≤ 0.05, and fold change ≥ 1.2. Red dots represent upregulated genes; green dots represent downregulated genes. **C** KEGG enrichment of differentially expressed genes in HUVECs. **D**–**G** GO enrichment analysis of differentially expressed genes in normal and high glucose-induced HUVEC. Enriched GO terms are shown for cellular component (**E**), molecular function (**F**), and biological process (**G**)

The final dataset contained 45 to 51 million raw reads per sample (Supplementary table S1). Following the filtering of the raw reads, sequencing showed that the average sample RNA-Seq library resulted in 41 million clean reads with an average mapping rate of 90.83% when aligned to human genomes (Supplementary table S2). For each cell type, the biological duplicates yielded reproducible normalized gene expression results showing Pearson correlation coefficients (R) in the following range: 0.90 < R < 0.99 (Supplementary Figure S1), indicating excellent experimental specificity and technical reproducibility. Next, a hierarchical cluster analysis of biological replicates was performed to visualize the experimental reproducibility of the selected genes (Fig. 5A).

Then we analyzed the transcription profiles of dermal fibroblasts in control and IL-7 conditions and screened for DEGs. Of the resulting genes, we selected those whose expression increased or decreased by more than twofold in IL-7-induced HDF, with a q value of ≤ 0.05. 99 DEGs (Fig. 5B) were obtained, including 55 upregulated and 44 downregulated DEGs.

Finally, the identified genes were subjected to KEGG pathway analysis and GO annotation. Significantly enriched KEGG pathways included cytokine-cytokine receptor interaction, rheumatoid, and IL-17 signaling pathways (Fig. 5C). Significantly enriched GO terms associated with the cellular component category included Extracellular space, extracellular region, and cell surface (Fig. 5D). The significantly enriched terms in the molecular function category were cytokine activity, growth factor activity, and chemokine activity (Fig. 5E). Finally, in the molecular function category, the immune response, cytokine-mediated signaling pathway, and cellular response to lipopolysaccharide were the most significantly enriched (Fig. 5F).

### IL-7 induced generation and secretion of ANGPTL4 in HDFs

**Fig. 6:**
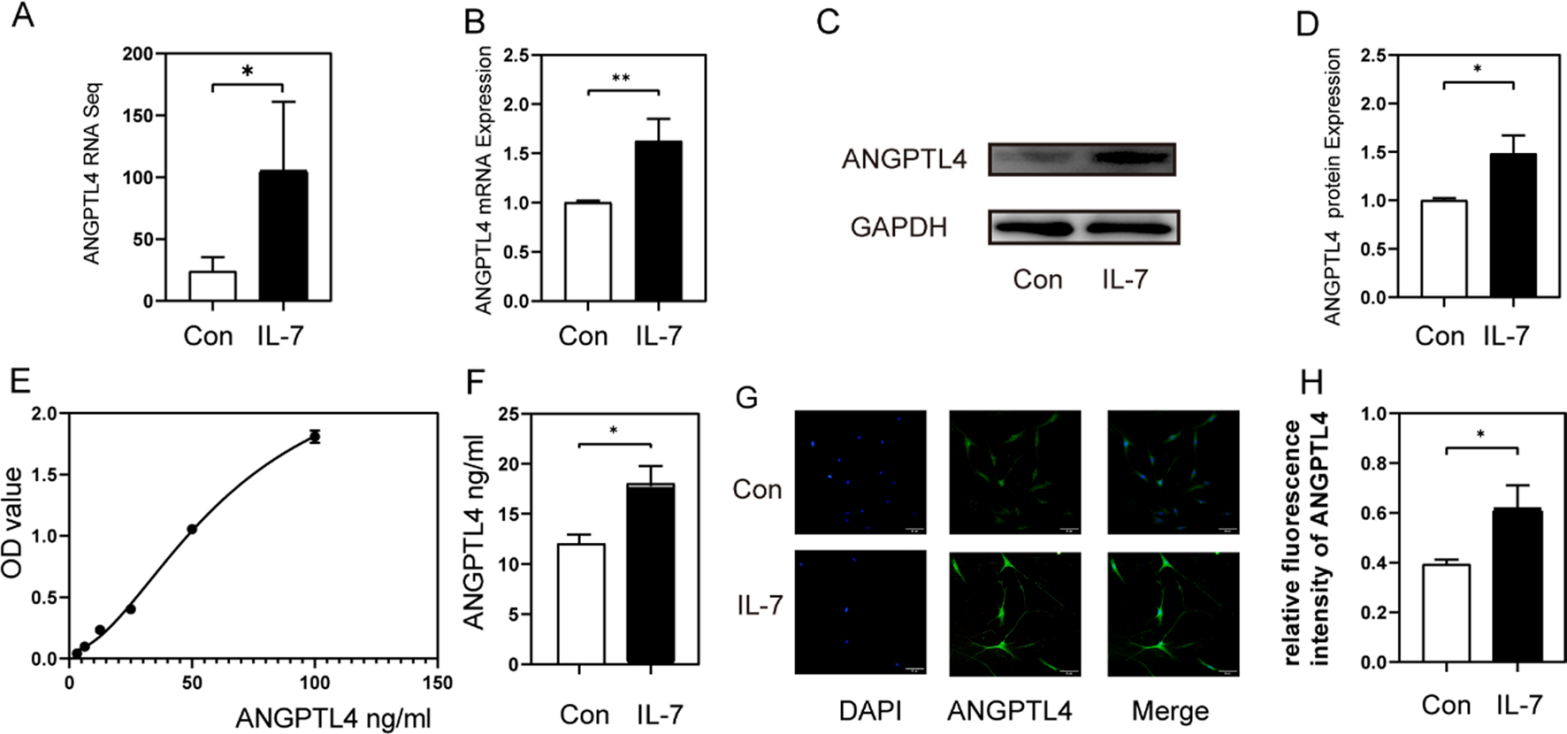
rhIL-7(40 ng/ml) induced generation and secretion of ANGPTL4 in HDFs. **A** RNA sequencing result of ANGPTL4 in HDFs in control and 40 ng/ml rhIL-7. **B** Relative ANGPTL4 mRNA expression in HDFs in in control and 40 ng/ml rhIL-7. **C**, **D** Representative western blotting and relative quantification analysis of GAPDH, ANGPTL4 in control and 40 ng/ml rhIL-7. **E** ELISA standard curve of ANGPTL4, **F** concentration of ANGPTL4 in the supernatant that cultured HDFs with 0 and 40 ng/ml rhIL-7. **G**, **H**: Representative immunofluorescent staining images and relative quantification analysis of ANGPTL4 (green) and DAPI (blue) in HDFs in control and 40 ng/ml rhIL-7. Scale bars, 50 mm. n = 3, *P < 0.05, **P < 0.01,

Among the upregulated genes found during RNA Sequencing, ANGPTL4 was selected for further studies (Fig. 6A). ANGPTL4 is a secreted protein that has been proven to inhibit angiogenesis in tumors. We found that IL-7 induced ANGPTL4 mRNA expression (Fig. 6B) and protein expression downregulation (Fig. 6C). Immunofluorescent staining assay showed that ANGPTL4 was significantly reduced in the cytoplasm (Fig. 6G). ELISA validated low levels of ANGPTL4 in the supernatant (Fig. 6E, F).

### Anti-ANGPTL4 treatment improved angiogenesis delayed by IL-7 and wound healing in STZ mice

**Fig. 7:**
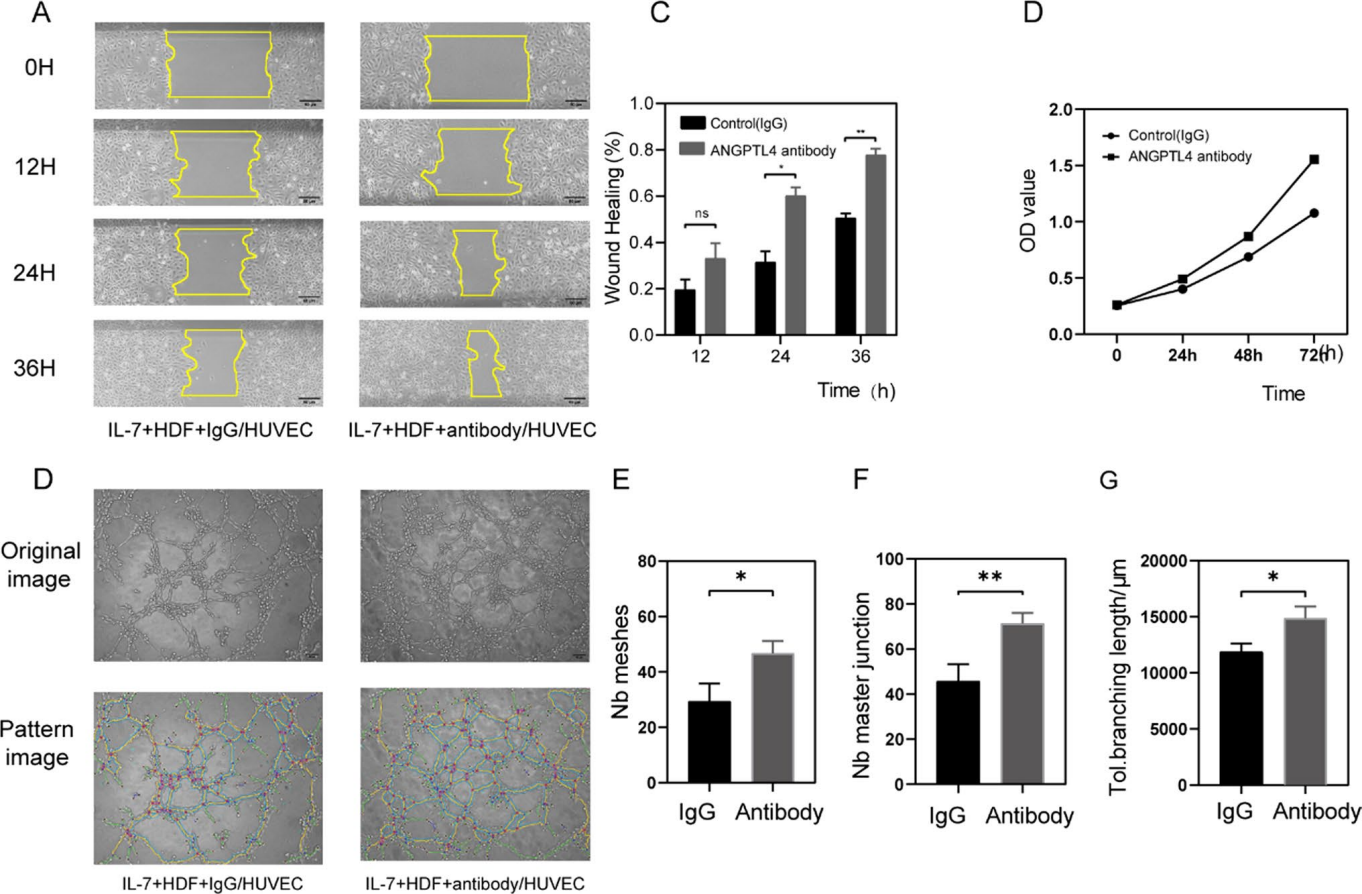
IgG (IgG group) and Human Angptl4-neutralizing monoclonal Antibody (Antibody group) were added to the 40 ng/ml-rhIL-7-induced HDF secretions in order to remove the inhibitory effect on HUVECs. **A**, **B** Representative scratch wound migration test image and analysis; HUVECs were treated in the rhIL-7-induced HDF secretions with IgG or Antibody for 0, 12, 24, 36 h; Scale bars, 50 mm. **C** CCK-8 assay, HUVECs were treated in the rhIL-7-induced HDF secretions with IgG or Antibody for days 0, 1, 2, 3. **D**–**G** tube formation assay image and analysis, **E** number of master junction **F** number of meshes, **G** total branching length, HUVECs were treated in the rhIL-7-induced HDF secretions with IgG or Antibody, 100ul/hole in 96-hole plate, Scale bars, 50 mm. n−3, *P < 0.05, **P < 0.01, ns, no significance

As shown in Fig. 3, angiogenesis was inhibited by IL-7-induced HDFs secretions. Then the anti-human ANGPTL4 antibody (0.1 mg/ml) was added to the secretion to neutralize ANGPTL4, while IgG (0.1 mg/ml) was added as a control. As expected, the inhibitory effect induced by ANGPTL4 was no longer visible. Anti-ANGPTL4 treatment improved proliferation (Fig. 7D) and migration (Fig. 7A) compared with IgG. Moreover, the treatment group exhibited a greater number of meshes and master segments and longer length of branch segments than the control group in the angiogenesis assay (Fig. 7D–G).

**Fig. 8:**
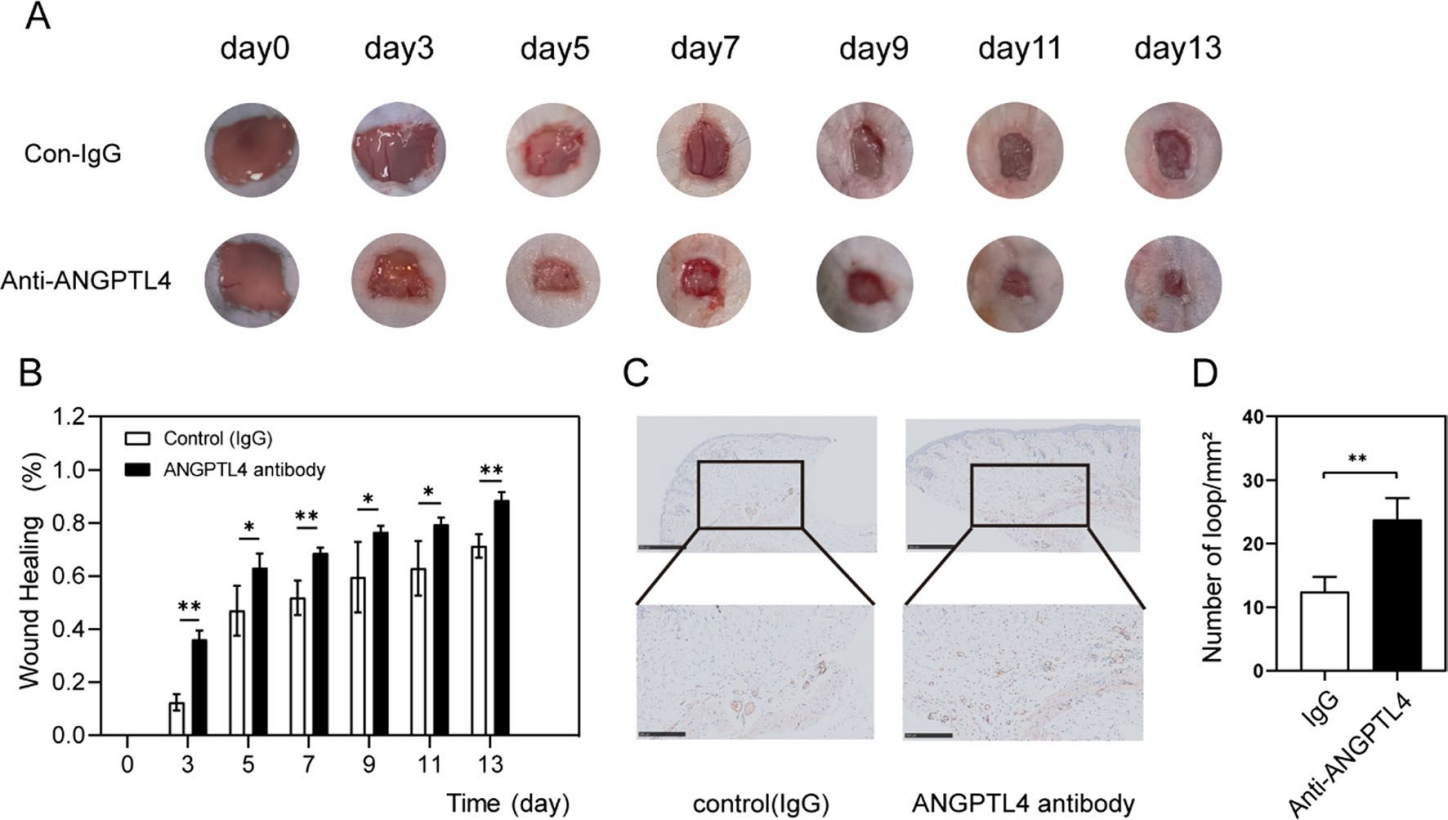
Full-thickness wounds were made on the two side of dorsum of STZ induced diabetic mice, and the wounds were treated locally with IgG (control side) and mouse Angptl4-neutralizing monoclonal Antibody (Antibody side) on days 0, 3, 5, 7, 9, 11 and 13 after injury. **A** Representative wound images at days 0, 3, 5, 7, 9, 11 and 13 during the healing process. **B** Wound healing area analysis showed accelerated healing in STZ induced diabetic mice treated with antibody and the healing is shown as the percentage of the initial wound area. **C** Levels of angiogenesis (vessel density) were evaluated by immunohistochemistry staining for CD31 in wound skin sections (day 11 post injury) from side of IgG side and antibody side, Scale bars, 250 mm. **D** Quantification of the vessel numbers in D is presented (n = 3, three views for each sample). n = 3, *P < 0.05, **P < 0.01

Finally, we tested the therapeutic effect of the anti-mouse ANGPTL4 antibody on diabetes mice. The diabetes mice were induced by STZ, which destroyed the pancreatic beta cell (Supplementary figure S3). Anti-mouse ANGPTL4 antibody (0.5 mg/ml) was added to one side, while IgG (0.5 mg/ml) was added as a control. As a result, the wound treated with neutralizing antibodies exhibited faster healing (Fig. 8B, C). The skin was cut for the immunohistochemical assay on day 11, and more angiogenesis was observed in the treatment group (Fig. 8D).

## Discussion

With the aging of the global population, it is expected that chronic wounds, especially diabetic ulcers, will continue to be a major medical, social, and economic challenge. (Sen 2021). Multiple pathophysiological mechanisms are involved in the occurrence of diabetic wounds in which angiogenesis plays a major role (Guo and DiPietro 2010). In our previous studies, fibroblasts, endothelial cells and keratinocytes were exposed to NG(5.5 mM) or HG (30 mM) medium for 24 h; RNA sequencing showed that IL-7 and IL-7R were significantly increased in fibroblasts (Zhang et al. 2021). Interestingly, we found that exogenous IL-7 delayed wound healing by inhibiting angiogenesis in normal mice. In vitro experiments revealed that IL-7-treated HDF secretions inhibited migration and angiogenesis of HUVEC. Further experiments showed that HDF ANGPTL4 secretion yielded an inhibitory effect, and the corresponding neutralizing antibody could block the inhibitory effect. Thus, our study revealed a potential signaling pathway in diabetic wound healing and provided a novel treatment for this patient population.

IL-7 is a growth-promoting factor for B-cells and T-cells, and several researchers have found that IL-7 could influence wound healing. Annie et al. found that the expression levels of IL‑7 were increased in healing chronic wounds (Bartlett et al. 2016). Osamu et al. documented that adding exogenous IL-7 reduces the production of collagen I and fibronectin (Yamanaka et al. 2006). In our study, RNA sequencing suggested IL-7 and IL-7R were altered in fibroblasts. We substantiated that IL-7 and IL-7R were increased in the skin of diabetic mice and fibroblasts treated with high glucose. This finding suggested that the IL-7-IL-7R signal pathway was activated under high glucose conditions. To observe the effect of IL-7, we used rMuIL-7 to treat normal mice. Interestingly, we found that rMuIL-7 inhibited angiogenesis in normal mice skin. Impaired angiogenesis leads to insufficient hypoxia and perfusion (Okonkwo and DiPietro 2017). Thus, we presumed that IL-7 could delay wound healing by inhibiting angiogenesis.

Angiogenesis refers to the formation that new blood vessels from the development of existing capillaries or post-capillary veins, mainly including vascular basement membrane degradation, vascular endothelial cell proliferation, and migration (Risau 1997). Given that endothelial cells play a central role in angiogenesis in response to angiogenic stimuli (El-Kenawi and El-Remessy 2013), rhIL-7 was added to stimulate HUVEC. However, the proliferation, migration and tube formation of HUVEC showed no difference with the control group. This finding suggested that IL-7 yields no direct effect on angiogenesis. Indeed, it remains unclear whether IL-7 has other effects on endothelial cells, warranting further research.

Next, we found that endothelial cells expressed more IL-7 than fibroblasts. Besides, IL-7R expression was higher in fibroblasts than endothelial cells under NG or HG conditions, and HG increased IL-7R expression of fibroblasts. We hypothesized that fibroblasts and endothelial cells were intricately correlated. It is well-established that wound healing is highly intricate and involves a sequence of signals and responses from fibroblasts, platelets, endothelial, epithelial, and immune cells (Guo and DiPietro 2010). The crosstalk between these signals is based on cytokines, such as TGF-β, VEGF, and TIMP-1 (Liu et al. 2008; Belvedere et al. 2020; Amiri et al. 2022). Fibroblasts play an essential role in tissue growth, development and repair, secreting extracellular matrix (ECM), including collagen, fibronectin and laminin (Hinz 2007). Growing evidence suggests that fibroblasts play a major role in governing angiogenesis (Tomasek et al. 2002). We hypothesized that IL-7 stimulated fibroblasts and fibroblasts secreted inhibitors that restrained proliferation, migration and tube formation of HUVEC. Accordingly, rhIL-7 was used to culture HDF for 24 h, and the supernatant was collected to stimulate HUVEC, inhibiting proliferation, migration and tube formation of HUVEC.

Next, we sought to explore the downstream signal transduction pathway. HDF was cultured in NG with 0 or 40 ng/ml IL-7 for 24 h, and total RNA was extracted for RNA sequencing procedures. The most enriched KEGG pathways were rheumatoid and IL-17 signaling pathways. It has been reported that expression of IL-7 and its receptor are increased in rheumatoid arthritis synovial fluid and fibroblasts, compared to normal cells (Pickens et al. 2011). Moreover, the levels of IL-17 and IL-20 are elevated in DB wounds (Finley et al. 2016). Accordingly, the effect of IL-7 and IL-7R in rheumatoid arthritis is worth exploring. Moreover, the relationship among IL-7, IL-17 and IL-20 in wound healing warrants further research. It has been reported that IL-7R is associated with JAK3, activating PI-3K and AKT (Jiang et al. 2005). However, our RNA sequencing results yielded no change in JAK3 or PI-3K, (Supplementary table S3) suggesting that post-translational modification may be involved.

Among upregulated and downregulated genes, ANGPTL4 was selected for further studies. ANGPTL4 is widely acknowledged for its role in lipid metabolism. Takada et al. found that ANGPTL4 inhibited angiogenesis as a tumor suppressor (Okochi-Takada et al. 2014). Guo showed that high glucose phosphorylated AKT and improved ANGPTL4 protein expression (Guo et al. 2020). Yang et al. substantiated that ANGPTL4 inhibited angiogenesis by its carboxyl terminus (Yang et al. 2008). Accordingly, we verified that the mRNA and protein expression of ANGPTL4 were increased in HDF cultured by rhIL-7. Since ANGPTL4 is a secreted protein, ELISA was used to quantify the concentrations of ANGPTL4 in the supernatant. These results were consistent with the RNA sequencing results. Based on these results, we conjectured that IL-7 stimulated HDF secretion of ANGPTL4, which could inhibit HUVEC angiogenesis. Next, the human Angptl4-neutralizing monoclonal antibody was applied to neutralize ANGPTL4, and the inhibitory effect was no longer visible. Finally, we treated STZ-induced diabetic mice with mouse Angptl4-neutralizing monoclonal antibody. Compared with the IgG group, neutralizing antibodies promoted angiogenesis and wound healing.

In summary, our present study corroborated that high glucose activates the IL-7- IL-7R signal pathway of HDF and promotes HDF secretion of ANGPTL4, which inhibits the angiogenesis of HUVEC. Given that IL-7 plays a role in the immune system, the diabetes chronic wound may involve immune dysfunction. Further studies on the receptor and post-receptor signaling events are warranted to uncover the mechanisms associated with pathological neovascularization in chronic diabetic wounds.

## Supplementary Information

Below is the link to the electronic supplementary material.

Supplementary file1 (DOCX 676 kb)

## Data Availability

The original data presented in the study are included in the article/Supplementary Material. Further inquiries can be obtained from the corresponding authors.
