# Supplementary material for: High glucose-induced IL-7/IL-7R upregulation of dermal fibroblasts inhibits angiogenesis in a paracrine way in delayed diabetic wound healing
Source: J Cell Commun Signal. 2023 May 22;17(3):1023–38. doi: 10.1007/s12079-023-00754-x (PMC10409704; doi:10.1007/s12079-023-00754-x)
Supplement: Supplementary file 1 — Supplementary file1 (DOCX 676 kb) [file 12079_2023_754_MOESM1_ESM.docx]

Supplementary Table1: Raw reads per sample

| Sample Name | N Read Num | Adapter Read Num | Low Quality Read Num | Clean Read Num |
| --- | --- | --- | --- | --- |
| HDF_con_1 | 2999116 | 2064606 | 1044448 | 39393746 |
| HDF_con_2 | 2982604 | 3447886 | 987832 | 38973594 |
| HDF_con_3 | 3299130 | 3805746 | 1090758 | 43380878 |
| HDF_IL7_1 | 1957070 | 3450946 | 700314 | 44624984 |
| HDF_IL7_2 | 466890 | 1300578 | 68 | 45559198 |
| HDF_IL7_3 | 418024 | 1716928 | 58 | 45191724 |

Supplementary Table2: Comparison to human genomes

| Sample Name | Total Clean Read | Total Mapping Genome Ratio | Uniquely Mapping Genome Ratio |
| --- | --- | --- | --- |
| HDF_con_1 | 39.39 | 90.47 | 85.34 |
| HDF_con_2 | 38.97 | 89.93 | 84.83 |
| HDF_con_3 | 43.38 | 89.77 | 84.67 |
| HDF_IL7_1 | 44.62 | 91.37 | 86.00 |
| HDF_IL7_2 | 45.56 | 92.31 | 86.92 |
| HDF_IL7_3 | 45.19 | 91.10 | 85.81 |

Supplementary Table3: Change of PI-3K-AKT and JAK-STAT pathway

| Gene ID | Gene Name | HDF_IL7_1 TPM | HDF_IL7_2 TPM | HDF_IL7_3 TPM | HDF_con_1 TPM | HDF_con_2 TPM | HDF_con_3 TPM |
| --- | --- | --- | --- | --- | --- | --- | --- |
| 207 | AKT | 97.76 | 97.88 | 107.13 | 104.38 | 105.98 | 112.81 |
| 3716 | JAK1 | 91.66 | 89.14 | 90.81 | 97.36 | 93.07 | 103.26 |
| 3717 | JAK2 | 3.39 | 3.24 | 3.01 | 3.71 | 3.67 | 3.71 |
| 3718 | JAK3 | 0.45 | 0.43 | 0.35 | 0.49 | 0.6 | 0.49 |
| 5291 | PIK3CB | 12.12 | 12.47 | 11.58 | 12.33 | 12.57 | 12.2 |
| 6772 | STAT1 | 135.05 | 117.79 | 182.01 | 173.61 | 133.43 | 217.54 |
| 6774 | STAT3 | 33.16 | 32.4 | 35.22 | 37.68 | 37.31 | 40.65 |
| 6776 | STAT5 | 2.5 | 1.81 | 2.14 | 3.4 | 2.18 | 3.63 |

Supplementary figure1


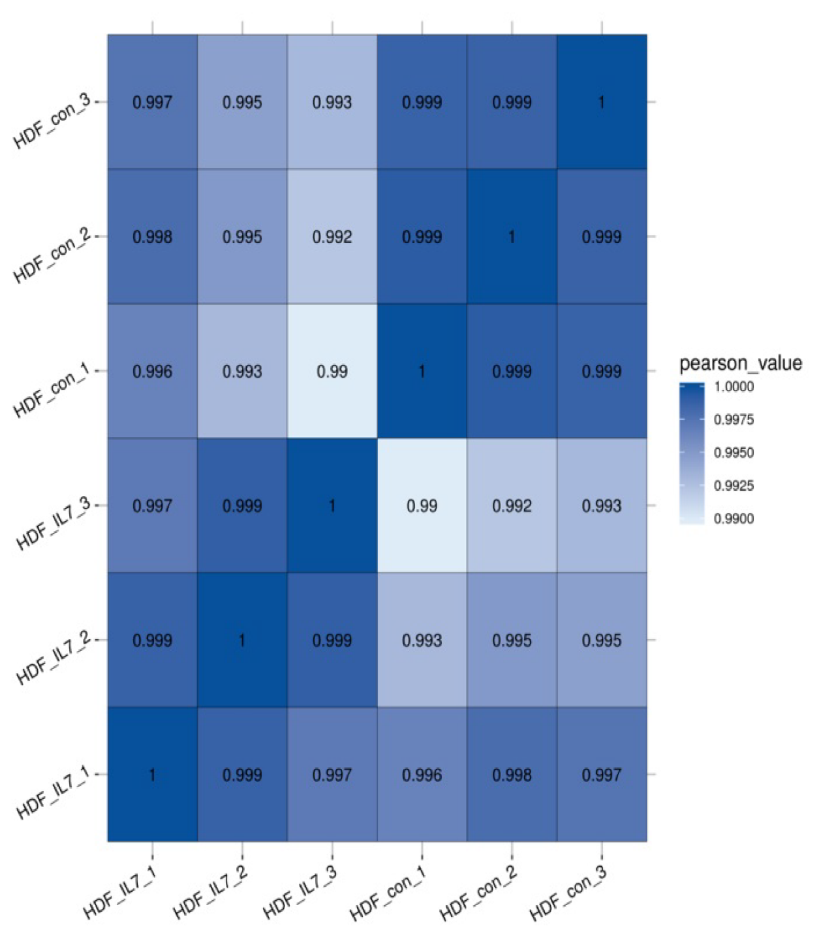


Supplementary figure2

Normal C57BL/6J Diabetic mice
